# The World’s Columbian Congress

**Published:** 1893-09

**Authors:** 


### EDITORIAL.

### The World's Columbian Dental Congress.

This great convocation, for which preparation has been in progress for more than two years, is now of the past, and it is the work of history to record the results. Its opening session began on the morning of Monday, August 14th, 1893, at 11 o'clock, and continued its general session for one hour and a half each day until the following Saturday.

At the general session each day there was one or two papers of general interest presented, and either then briefly discussed or the discussion was referred to the appropriate section—the larger number of the papers were thus referred—the general session afforded but very brief time for discussion. There were two evening sessions of the general meeting, for the presentation of papers, for the illustration of which the Micro-Lantern was used. One evening was occupied by Dr. R. R. Andrews, of Cambridge and W. X. Sudduth, of Minneapolis; the second evening was occupied by Dr. George Cunningham, of England; these were occasions of very great interest. The attendance at these general sessions was from four to five hundred. The greater proportion of the work in the way of reading papers and discussion upon them was had in the various sections, of which there were eight, viz; first, Anatomy and Histology; second, Etiology, Pathology and Bacteriology; third, Chemistry and Metallurgy; fourth, Therapeutics and Materia Medica; fifth, Dental and Oral Surgery; sixth, Operative Dentistry; seventh, Prosthesis and Orthodontia; eighth, Education, Legislation and Literature.

The time assigned to these meetings of the sections was from 2:30 to 5:30 P. M., and they were usually well attended, some of them full to overflowing. The papers read and the discussions were of great interest, the latter participated in by a very large number of the members. It may be said that all the papers were good, quite a goodly proportion were better, and a few were best. A few of the members expressed some disappointment that there were not more superior papers, and of them, as a whole, it may with truth be said, that there was very much for thought and consideration in the papers; and this perhaps, will be more apparent when the transactions are published and the papers can be carefully read and studied.

In several of the sections, especially in the sections of Surgery, Prosthesis and Orthodontia, many instruments and things were exhibited in illustration of the points made in the papers and discussions. To all, these were instructive, and especially so to those the more directly interested in any given line. Far more work was done in the aggregate by the sections than could have been accomplished by devoting the whole time to general sessions, and after all, it would have been a very great attainment if all the work done could have been enjoyed by all the members present, instead of only a comparatively few being able to make the work on any given subject their own property.

The papers presented by our brethren from other countries possessed much merit, and the profession of the United States owes them a debt of gratitude for that which they brought to us. If there was disapointment in any one thing more than another it was, perhaps, that more of our brethren from abroad were not present. It was the hope and anticipation of many that a large number would be here from other countries, and not only add to the scientific work of the body, but would in a good measure add to the social features of the occasion; but in this respect there was a good degree of success. The opportunity for social enjoyment was, by the arrangement which had been provided, very liberal. This meeting having no antecedent and not to have a succeedant, of course, had very little of parliamentary or routine work, so that, about all the time was devoted to scientific work or to social amenities, so that, if any one failed to meet and become acquainted with almost everybody else it was his own fault.

The attendance though not as large as was anticipated six months before the meeting, was larger, perhaps, than any one dared to anticipate during the last three months preceding the meeting. The financial trouble that has spread over our country, and, indeed, has prevailed to a greater or less extent throughout all the civilized countries of the world for the last few months, undoubtedly, was the occasion of many being absent. This could not have been foreseen nor provided against, and the only thing, in a great many instances, was to yield to the force of circumstances. There were registered a little over thirteen hundred names, (the exact number I have not just at hand) this is a larger number of dentists than was ever together before in the world, probably, more than twice as many. Perhaps, not much over half the number registered were in the hall at the general meeting, at any one time. The aggregate number attending the sections was considerably larger, perhaps than was present at the general meeting; this occurred because many would gravitate to the sections where matters were under consideration in which they were especially interested.

In the exhibition of materials, instruments, appliances, etc., there was a large array of things new and old, of things good, bad and indifferent, some of exceeding interest, all perhaps of interest to some.

Clinics were held on five days, and it was a very frequently expressed wish that they could have been more successful; they were held in quarters far too small and not sufficiently accessible; they were in rooms in the top of the building, immediately under the roof, and were always uncomfortably warm. On account of inadequacy of room there was a constant surging, buzzing crowd, in which no operator could do himself justice, and, indeed, no one should have consented to operate under such circumstances. Who was responsible for this arrangement we do not know, but, doubtless, if it were to be arranged again better facilities would be afforded. Probably not fifty persons gained anything of real value from the clinics, and certainly those who attempted to operate fell much short of their best efforts.

In considering the regular attendance at all the work of the congress it may be said that it was quite as good and even better than was anticipated by many; there were so many outside attractions. Chicago, with all its attractions, and they are innumerable, and above all The World's Fair; these things could not be resisted by a great many who were in attendance. The remark was often made "We will have an opportunity to attend dental meetings in the future, but probably never again to witness a World's Fair like this," and so it was, a large number were every day absent from the meetings, and either wandering in Chicago or visiting The World's Fair. As to what might have been accomplished if in better financial conditions, imagination or guessing can only portray; had all things been favorable, as they were a year ago, there would undoubtedly have been present from two to three thousand members of the dental profession; but considering all circumstances no one familiar with all that was accomplished can hardly be otherwise than gratified.

The transactions will be issued within a few months, when the profession will have an opportunity to calmly consider the work that has been done, and no doubt great good will result.

